# Neuroimmunology of CNS HIV Infection: A Narrative Review

**DOI:** 10.3389/fneur.2022.843801

**Published:** 2022-06-14

**Authors:** Ana-Claire Meyer, Alfred Kongnyu Njamnshi, Magnus Gisslen, Richard W. Price

**Affiliations:** ^1^Department of Neurology, Johns Hopkins University School of Medicine, Baltimore, MD, United States; ^2^Neuroscience Laboratory, Faculty of Medicine and Biomedical Sciences, The University of Yaoundé I, Brain Research Africa Initiative (BRAIN), Yaoundé, Cameroon; ^3^Department of Infectious Diseases, Institute of Biomedicine, Sahlgrenska Academy at University of Gothenburg, Gothenburg, Sweden; ^4^Department of Infectious Diseases, Region Västra Götaland, Sahlgrenska University Hospital, Gothenburg, Sweden; ^5^Department of Neurology, University of California San Francisco (UCSF), San Francisco, CA, United States

**Keywords:** Africa, HIV, inflammation, central nervous system (CNS), cerebrospinal fluid (CSF), neuroimmunology, antiretroviral therapy (ART)

## Abstract

This short review provides an overview of the interactions of human immunodeficiency virus type 1 (HIV), immune and inflammatory reactions, and CNS injury over the course of infection. Systemic infection is the overall driver of disease and serves as the “platform” for eventual CNS injury, setting the level of immune dysfunction and providing both the HIV seeding and immune-inflammatory responses to the CNS. These systemic processes determine the timing of and vulnerability to HIV-related neuronal injury which occurs in a separate “compartment” with features that parallel their systemic counterparts but also evolve independently. Direct CNS HIV infection, along with opportunistic infections, can have profound neurological consequences for the infected individual. HIV-related CNS morbidities are of worldwide importance but are enhanced by the particular epidemiological, socioeconomic and environmental factors that heighten the impact of HIV infection in Africa.

## Introduction

HIV is a retrovirus taxonomically grouped in the genus Lentivirus (1) that entered the human population through multiple zoonotic infections from simian immunodeficiency virus–infected nonhuman primates (2). Its double-stranded RNA genome is more complex than many other retroviruses, and in addition to structural genes it contains several regulatory and accessory genes that contribute to its detailed life cycle, protracted course and pathological consequences. While all viral proteins presumably play a role in the character of infection, some have been singled out as particularly important in determining the character of CNS infection and its consequences. These include, for example, the *env* (envelope) gene that determines T-cell or macrophage tropism (T- or M-tropism) that dominate in different phases of CNS infection (3); Likewise, the accessory genes, including *tat*, may contribute to neurotoxicity (4). HIV is also subdivided into four groups with several subtypes or clades (5). The importance of group and clade variations for neurological complications, particularly those related to direct CNS infection, remains incompletely defined (6). This review focuses on emerging concepts in the neurobiology of more “direct” CNS complications of HIV-1 infection, particularly HIV-associated dementia (HAD) and, by inference, also milder cognitive impairments.

## Clinical Background

### HIV Epidemiology and Impact in Africa

Since its onset, the HIV pandemic has disproportionately impacted the African continent. While the first case definitions for AIDS were developed in 1982 (7), by the end of 2001 there were 40 million people living with HIV (PLWH), of whom 28.5 million (71%) were located in sub-Saharan Africa, at that time without access to antiretroviral therapy (ART) (8). While ART first became available to resource-rich countries in the 1990's, it took another decade of grass-roots political advocacy before ART first became more widely available in Africa through the United Nations Global Fund and the US President's Emergency Plan for AIDS Relief (9). Over the subsequent two decades, there has been tremendous progress in scaling up HIV care and treatment, and in 2021, 27.5 million PLWH globally were taking ART.

However, there remain important gaps. The prevalence of HIV in Africa varies widely among countries, from a low of <0.1% in Algeria and Egypt to more than 19% in South Africa, Botswana, Lesotho, and Eswatini (10). There remain 10.2 million PLWH who are not on HIV treatment, and in 2020 there were 1.5 million new HIV infections and 680,000 deaths (11). In sub-Saharan Africa, women and children are particularly vulnerable; in sub-Saharan Africa, women aged 15–49 make up 52% of new infections though they only represent 24% of the population. Older children (age 5–14 years) make up two-thirds of those not on treatment, and only 40% of children living with HIV had suppressed viral loads, as compared to 67% of adults (11).

### CNS Disease in Africa

CNS complications of HIV are important causes of morbidity and mortality in Africa, and indeed globally (12). Descriptive epidemiology of HIV-associated CNS disease in Africa is limited by the availability of neurologists and advanced diagnostics such as computed tomography (CT), magnetic resonance imaging (MRI), and cerebrospinal fluid (CSF) analysis (13). Thus, many studies and clinical management decisions rely on syndromic clinical diagnoses with limited diagnostic precision, depending on the local resources. However, CNS opportunistic infections (OIs) are clearly common causes of hospitalization and may cause approximately 20% of deaths (14, 15). For disorders such as HIV-associated dementia (HAD) and, by inference, also milder cognitive impairments, diagnostic precision is even more limited.

Estimates of the prevalence of HIV-associated cognitive impairment have varied widely across the continent but are comparable to other world regions (16, 17) and have generally decreased as ART became more widely available (18–20). The prevalence of mild impairment was reported to be between 40 and 55% and moderate to severe impairment between 3 and 25% in two large multi-country cross-sectional and cohort studies using comprehensive neuropsychological test batteries in the African continent [the AIDS Clinical Trials Group 5199 (17, 21) and the African Cohort Study (22)], and in several larger studies from South Africa (23), Malawi (24), Tanzania (25) and Zimbabwe (26). Cognitive development is also impacted in pediatric HIV, where infants and young children with HIV do not perform as well as their HIV-exposed or HIV uninfected peers (27–30).

The variation in estimates of HIV-associated cognitive impairment in across Africa may be due in part to the use of tests with limited cultural validity, lack of well-matched norms and relying on screening tools with limited sensitivity and specificity when resources for neuropsychological testing are limited (31–33). In particular, the clinical relevance of mild impairment on neuropsychological tests in African populations is unclear (31) and test performance is impacted by literacy (22) and education level (23). HIV-uninfected individuals often perform poorly on tests (22), there is significant between country variation in normative data (21), and particularly among older individuals, there may be no group level differences observed between HIV-infected and -uninfected individuals (34, 35).

### Pathophysiology: HIV Neuroimmune-Virus Interactions and Their Impact on the CNS

**Figure 1:**
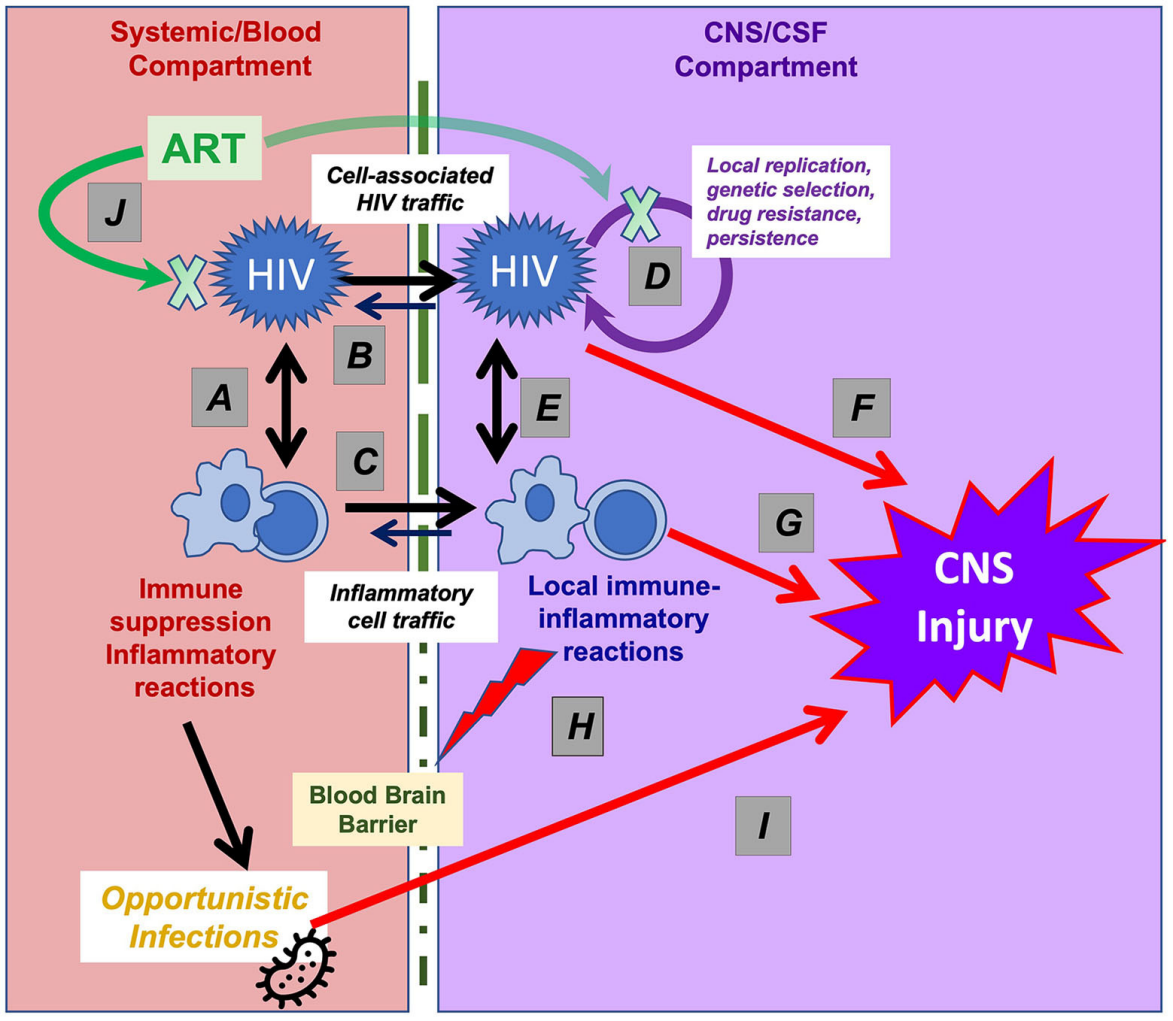
Interactions of HIV and immune-inflammatory responses in systemic and CNS infection. This simplified schematic outlines the systemic and CNS viral-immune interactions that determine the immunopathogenesis of CNS injury. Systemic interactions (shown in the left part of the figure) establish the foundation for CNS vulnerability that are partly echoed by interactions within the CNS (right part of the figure), though with important differences. **(A)** HIV targets CD4+ T lymphocytes (and to a lesser degree myeloid cells) in which viral replication both sustains viremia and establishes long-term viral persistence, leading to gradual T-cell loss and immunosuppression and to lifelong infection. Virus-induced T-cell activation, in turn, enhances viral replication and dissemination. **(B)** Systemic viremia is the source of CNS HIV infection, beginning early in infection, likely mainly via infected T cells that migrate through the blood-brain barrier (BBB, depicted by vertical dotted line). **(C)** Cells important to the CNS inflammatory response also derive from blood sources; these include CD4+ and CD8+ T cells and macrophages that elaborate cytokines and other signaling and toxic molecules that contribute to the compartmentalized CNS inflammatory response within the CNS and are reflected in CSF. **(D)** HIV can replicate locally within these migrating CD4+ T cells and macrophages sustaining a genetically independent infection and perhaps establishing a longer-lived second viral reservoir within the CNS. **(E)** The interaction of the local HIV infection with “imported” inflammatory cells and native CNS cells (including astrocytes and microglia) establish an independent inflammatory milieu that evolves over the course of disease and is particularly heightened in HAD/HIVE. **(F)** Both HIV gene products and **(G)** host inflammatory reactions likely contribute to ‘indirect’ CNS injury. **(H)** inflammatory reactions can disrupt the blood-brain barrier, further exacerbating this injury. **(I)** CNS OIs may involve a similar pathway, first with loss of systemic immune surveillance allowing entry or activation of pathogens that then invade the CNS and cause neurological disease by direct injury or through a local inflammatory response. **(J)** ART reverses or mitigates all of these processes. By suppressing HIV replication, treatment fosters a variable degree of CD4+ T cells restoration and partial reversal of these pathological processes. Abrupt restoration of immunity may lead to robust local inflammation and the immune restoration inflammatory response (IRIS) with exacerbation of neurological symptoms and signs. The blood-brain barrier variably impedes CNS concentrations of certain drug components of ART, delaying or reducing local antiviral effects and, in rare cases, contributing to the development of neurosymptomatic CSF escape despite systemic viral suppression.

Among the viruses considered in this collection, HIV likely has the most complex and intimate interactions with the immune system and inflammatory responses, both outside (i.e., systemically) and within the CNS. In both systemic and CNS compartments these interactions change over the long course of chronic infection (36, 37). Figure 1 diagrams these interactions, dividing the ***systemic*** (left) from ***CNS*** (right) processes. The elements in these two compartments interact, and more particularly, systemic HIV disease serves as the *foundation* for the CNS complications in several aspects. It establishes the conditions of immunosuppression and immune activation that underlie CNS vulnerability (37–40), and, more directly, supplies the key elements of neuropathogenesis, including HIV invasion and major blood-derived cells involved in CNS immune-inflammatory reactions. However, while CNS virus-immune interactions partially echo those occurring systemically, there are important differences, with the CNS interactions being highly compartmentalized despite these systemic origins (36).

In both systemic and CNS compartments the interactions of HIV and immune reactions evolve in important ways over the protracted course of chronic untreated infection. While the CNS infection echoes its systemic counterpart, it also diverges in important details, including in virus populations and particular inflammatory profiles (36, 41). If unchecked by ART this chronic course may be complicated by a range of disorders afflicting the brain, including major OIs and direct neuropathic HIV CNS infection (42, 43). Because of space constraints, this review omits detailed discussion of CNS OIs as well as disorders of the spinal cord and peripheral nervous system (PNS) that may be impacted by similar disease processes (44).

#### Progressive Systemic HIV Infection: Prerequisite and Facilitator of Major AIDS-Associated CNS Diseases

A number of the features of systemic HIV infection are important for the development of CNS HIV infection and disease. Ultimately, these stem from the fact that CD4+ T lymphocytes and, to a lesser extent, macrophages and related myeloid cells, are the main cellular targets of HIV infection (45–50). This targeted infection leads to progressive immunosuppression and also to a state of enhanced immunoactivation, with both contributing to CNS disease consequences (51–54). HIV infection is chronic and persistent, but importantly mitigated by ART. It remains, however, a major challenge to therapeutic cure efforts (55, 56), and stopping ART almost inevitably leads to a return of viremia accompanied by CSF viral rebound (57, 58).

Complications of HIV vary with the stage of systemic disease progression, most easily assessed by the blood CD4+ T lymphocyte count (38, 59, 60). AIDS is defined by the development of major OIs (and, in parallel, including HIV-associated dementia, *HAD*) or by a CD4+ count falling below 200 per μl (61).

CNS OIs develop when there is loss of systemic immune surveillance that allows certain organisms to escape a latent or quiescent presence in the body (e.g., JC virus or *Toxoplassma gondii*) or to evade defenses that would otherwise prevent systemic dissemination (e.g., *Cryptococcus neoformans*); this is followed by subsequent failure of these same defenses to eliminate these pathogens within the CNS. The spectrum of common CNS OIs is relatively circumscribed and involves organisms of relatively low pathogenicity that are otherwise readily contained or prevented by T-cell/macrophage defenses in the normal host. In Africa where *M. tuberculosis* is common in the community, HIV infection also enhances susceptibility even if this organism isn't readily classified as strictly “opportunistic” and is more common even at CD4 counts above those defining AIDS (15). However, the common CNS OIs generally occur at <200 CD4+ cells/μl (43). We emphasize this well-known susceptibility here because this also defines the susceptibility to HIV encephalitis (HIVE) and HAD which usually develops below this T-cell threshold (62), indicating that a similar level of immunosuppression is a prerequisite. In a sense HIVE might also be viewed as a CNS OI in which the same virus “creates the opportunity” through chronic systemic infection of CD4+ T cells before it can then “opportunistically” cause encephalitis. However, as discussed below, this does not apply to overall susceptibility to CNS HIV infection *per se*, but to “invasive” neuropathic encephalitic infection. In fact, low-grade HIV-1 meningeal infection is a common feature of systemic HIV infection that develops early in its course (37). The CNS is exposed to HIV very early in systemic infection, though it is often silent or accompanied by headache, fatigue or other unspecific symptoms. More rarely, acute encephalitis may develop during primary infection, likely involving an immunological pathogenesis (63). Over the course of chronic infection, milder neurocognitive impairment may develop and relate to low-grade forms of the viral and immunological processes that underlie HAD/HIVE, though these connections remain to be more precisely defined.

#### Systemic Origin of the Elements of CNS Infection

In addition to providing the background foundation and necessary level of immunosuppression for OIs and HIVE, systemic infection more directly underlies HIV CNS disease by providing both the invading virus and principal inflammatory cells that react to infection and contribute to immunopathology.

Most probably, HIV seeding of the CNS occurs *via* trafficking infected CD4+ T cells rather than by more direct virion penetration of the blood-brain barrier (64, 65). Infected cells entering the CNS can clonally expand and release (clonal) virus; this can then lead to further infection of susceptible cells, amplifying infection and establishing local replication (66). During later stage infection monocytes may also enter the CNS (65–70). This later CNS infection may be more *compartmentalized* with more notable evolution of virus populations independent of those examined in blood. Uninfected CD4+ T cells and monocytes may also enter the CNS contributing to amplified infection. This can also lead to local CNS HIV persistence after treatment, though, this has been less clearly defined, including the types of cells and anatomic locations, state of viral expression and mechanisms of replication control.

#### Dynamics of CNS Infection With Disease Progression: Transition From Meningitis to Encephalitis

A central feature of CNS HIV infection is its changing character with systemic disease progression. This includes shifts in the relation of CSF and blood viral populations (71–73), changes in the accompanying inflammatory profiles (36) and eventual shift in the main anatomic site of productive infection from the leptomeninges to the brain in some individuals. In the earlier phase of infection when blood CD4+ T cell levels are above 200 per μl, the leptomeninges are the most conspicuous location of chronic CNS HIV-1 infection so that a clinically silent aseptic meningitis is frequent. This infection is largely “equilibrated” with CSF HIV RNA concentrations maintained at levels near 10 percent of those in blood (37, 74, 75), and CSF and blood populations are genetically similar (76), presumably because of continuous and fresh virus traffic from blood to CSF. When CD4 cells fall below 50/μl, the ratio of CSF to blood virus deceases to near 1% blood HIV RNA levels as CSF pleocytosis also diminishes, consistent with a relation between CSF WBCs and viral load (77–80). The extent of penetration of infection into the brain parenchyma at this stage is uncertain, but if present it is largely clinically silent. Whether this early type of infection and inflammation is responsible for milder cognitive impairment is still not definitively established, though often presumed.

These relationships change in those who develop HIVE that presents clinically as subacute HAD (36). This condition usually develops after blood CD4+ cells fall below 200/μl and represents an extension of infection from meninges into the brain parenchyma. White matter abnormalities are usually prominent on MRI but gray matter also is frequently affected, particularly the basal ganglia (81–83). While inflammation in those without HIVE largely involves lymphocyte-related cytokines, as CD4+ T cell counts fall, macrophage-related inflammation increases. In those with overt HIVE there is augmentation of both lymphocytic and macrophage biomarkers (36). CNS viral populations in these individuals are more compartmentalized in relation to those in blood, and exhibit macrophage tropism (76, 84). While astrocytes can be infected by HIV, this is usually considered to be non-productive with limited gene expression; hence, their role in persistence and neuropathogenesis is still uncertain (85, 86). Importantly, neurons are not infected, and thus damage to neurons is largely or exclusively by “indirect” mechanisms, meaning that they are injured *from without* by signals and toxins released by neighboring cells rather than from direct effects of viral genes and their products expressed within these cells (87). Likely the external toxic signals are elaborated mainly from inflammatory cells, perhaps predominantly from macrophages and other myeloid cells. Late in infection HIVE also commonly disrupts the blood-brain barrier, further contributing to neuronal injury and dysfunction (36, 88, 89).

#### Impact of ART on CNS Infection and Disease

ART has had a profound effect on preserving CNS integrity, both in preventing HAD/HIVE development and in mitigating this CNS disease after it manifests (90, 91). This effect may be in part through preservation or restoration of immunity but mainly by more directly suppressing both systemic viremia and HIV replication within the CNS. As a result, HAD incidence is now markedly reduced and confined largely to those not receiving ART.

For individuals who present with HAD, having fallen through defects in the treatment network, ART can arrest and often reverse the severity of its impact, depending on the time frame of HAD development and treatment initiation. Diagnosis should be made quickly, and treatment begun rapidly. This is a setting in which both the antiviral potency and CNS penetration of the components ART regimens are likely important (92, 93). In some of treated individuals the degree of short- and long-term recovery can be remarkable.

#### CSF Escape

This term refers to situations in which the impact of ART on CNS HIV infection is relatively reduced compared to that on systemic infection, leading to *CSF HIV RNA levels exceeding those of plasma* (94–98). Three distinct types of CSF escape have been defined: *asymptomatic, neurosymptomatic* and *secondary*. The most important of these is n*eurosymptomatic* CSF escape in which ART-treated individuals present with new or progressive neurological deficits (96–100). Most often, in addition to symptoms and signs of CNS injury and dysfunction, there is CSF pleocytosis, elevated CSF neurofilament light chain protein (NfL) concentration, and neuroimaging abnormalities consistent with active CNS HIV infection. Neurosymptomatic escape overlaps with pathologically-defined CD8 encephalitis (101–103). In most cases a background of reduced treatment adherence and drug resistance, at times in combination with insufficient CNS penetration of component antiviral drugs, can be identified (96). This provides further support for the need for targeted treatment of CNS, at least in some settings. Inflammation and immunopathology may be an important mechanistic component in this setting in which CD4+ T cell counts are higher than in HAD/HIVE because of the disproportionate systemic efficacy of ART that fosters CD4+ cell recovery and suppresses systemic viremia. CNS HIV isolates often exhibit drug resistance, though not always. The main avenue of treatment is changing the ART regimen to a potent antiviral drug combination that includes component drugs to which the CSF/CNS virus is susceptible and also achieve therapeutic brain concentrations.

The other two forms of CSF escape are of less clinical importance. Asymptomatic escape is an incidental finding mainly in CSF cohort studies. It is characterized by detectable CSF HIV RNA in the presence of plasma viral suppression; CSF HIV RNA levels are usually low with little or no pleocytosis. By definition these individuals lack new neurological symptoms or signs (104, 105). Secondary escape entails a disproportional increase in CSF HIV RNA in association with another CNS inflammatory process (usually another CNS infection) that provokes local HIV replication through recruitment of activated lymphocytes. Treatment of the provoking infection leads to reduction of the CSF HIV RNA elevation (79).

#### CNS Persistence and Cure

Despite the effectiveness of ART in suppressing systemic and CSF HIV infection, it does not cure HIV. When ART is stopped, viremia and CNS replication re-emerge (106). Because of this intractable persistence of HIV, efforts are now underway to effect a systemic cure using a variety of strategies (55). There is precedent with bone marrow transplant using an HIV-resistant donor. In one well-studied case, not only was there no evidence of viral persistence systemically but also no trace of virus in CSF (107). More broadly it remains an open issue as to whether the CNS serves as an independent viral reservoir that might require CNS-targeted cure strategies.

## Conclusion

CNS HIV infection is a component of the “ecology” of HIV, an offshoot of systemic viremia that can lead to important morbidity and mortality. Fortunately, ART has a major impact on CNS infection and its effects, from Pre-Exposure Prophylaxis (PrEP) preventing initial infection, to early treatment of infection that likely reduces the CNS reservoir (91), to treatment of established HAD/HIVE. Thus, while additional interventions, including vaccines and cure efforts are welcome, widespread use of preventative and therapeutic ART continue to have a major impact on neurological disease in HIV and AIDS.

## Author Contributions

All authors listed have made a substantial, direct, and contribution to the work and approved it for publication.

## Conflict of Interest

MG has received research grants from Abbott, Baxter, Bristol-Myers Squibb, Gilead Sciences, GlaxoSmithKline, Merck, Pfizer, Roche and Tibotec, and he has received honoraria as a speaker and/or scientific advisor from Abbott/Abbvie, Amgen, Biogen, Bioinvent, Boehringer-Ingelheim, Bristol-Myers Squibb, Gilead Sciences, GlaxoSmithKline, Janssen-Cilag, MSD, Novocure, Novo Nordic, Pfizer, Roche and Tibotec. A-CM is a paid employee of Denali Therapeutics. The remaining authors declare that the research was conducted in the absence of any commercial or financial relationships that could be construed as a potential conflict of interest.

## Publisher's Note

All claims expressed in this article are solely those of the authors and do not necessarily represent those of their affiliated organizations, or those of the publisher, the editors and the reviewers. Any product that may be evaluated in this article, or claim that may be made by its manufacturer, is not guaranteed or endorsed by the publisher.
